# Osteonevus of Nanta: a case report in a combined melanocytic nevus^☆^^☆☆^

**DOI:** 10.1016/j.abd.2019.04.017

**Published:** 2020-03-19

**Authors:** Elaine Dias Melo, Patrícia Amaral Couto, Antônio Pedro Mendes Schettini, Carlos Alberto Chirano Rodrigues

**Affiliations:** a Service of Dermatology, Fundação de Dermatologia Tropical e Venereologia Alfredo da Matta, Manaus, AM, Brazil; b Service of Dermatology Surgery, Fundação de Dermatologia Tropical e Venereologia Alfredo da Matta, Manaus, AM, Brazil

**Keywords:** Heterotopic, Nevus, blue, Nevus, intradermal, Nevus, pigmented, Ossification, Osteoma

## Abstract

Secondary osteoma cutis is a phenomenon that may occur in several conditions. When it occurs in a melanocytic nevus it is named osteonevus of Nanta, an event considered uncommon and characterized by the presence of bone formation adjacent or interposed with melanocytic cells. There are reports of its occurrence in various melanocytic lesions, being more frequently associated with intradermal nevus. We report a case of osteonevus of Nanta in combined nevus, possibly the first description of this association.

## Introduction

Secondary skin ossification is a phenomenon that can occur in several conditions, such as pilomatricoma, basal cell carcinoma, acne, scars, mixed skin tumors, sites of inflammation and trauma.^1^ Its occurrence in melanocytic nevus, called osteonevus of Nanta, appears to be an uncommon event with an estimated incidence from 0.6% to 1.45% among pigmented tumors.^2^

The osteonevus of Nanta is characterized by the presence of adjacent bone tissue or interposed with melanocytic cells,^3^, ^4^ usually located on the upper trunk and with predilection for female.^2^

We report the case of a woman with exuberant lesion on the scalp, whose histopathological analysis was conclusive of osteonevus of Nanta, being possibly the first report of this association.

## Case report

**Figure 1:**
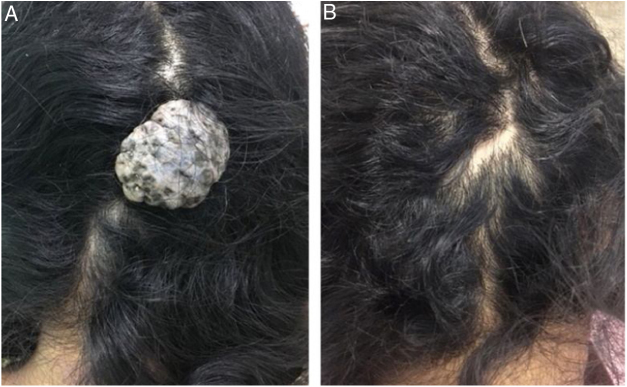
(A) Exophytic, pedunculated tumor of fibroelastic consistency, measuring 3.0 × 2.0 cm, on the occipital region; (B) appearance after surgical removal.

**Figure 2:**
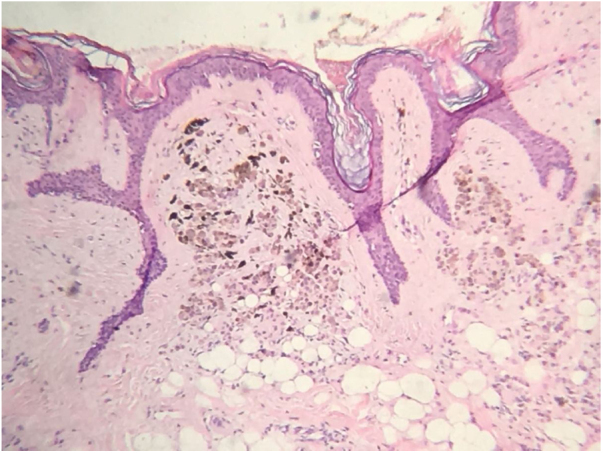
Diffuse proliferation of pigmented epithelioid melanocytic cells in the papillary dermis compatible with intradermal nevus (Hematoxylin & eosin, ×100).

A 38 year-old woman with an occipital lesion since she was born, which increased in the last 14 years, without associated symptomatology. At the examination, there was an exophytic, pedunculated, normocromic tumor with irregular surface and depressed areas with dark brown to blue pigmentation, fibroelastic consistency, measuring about 3 × 2 cm in diameter (Fig. 1). The patient had no comorbidities, but she claimed to be a user of illicit drugs. We performed surgical excision of the lesion for diagnosis and treatment, with fusiform excision and primary suture with simple stitches (Fig. 2).

**Figure 3:**
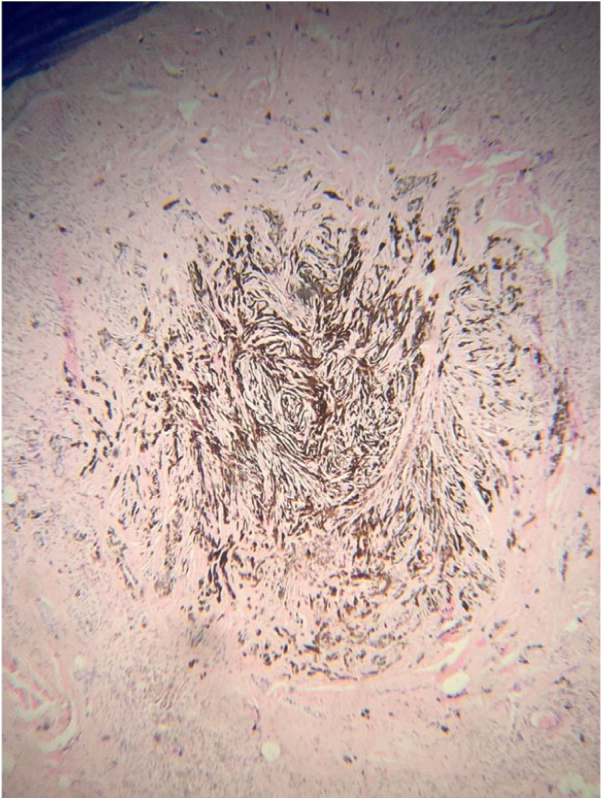
Spindle and dendritic pigmented cells distributed in bundles in the dermis (Hematoxylin & eosin, x400).

**Figure 4:**
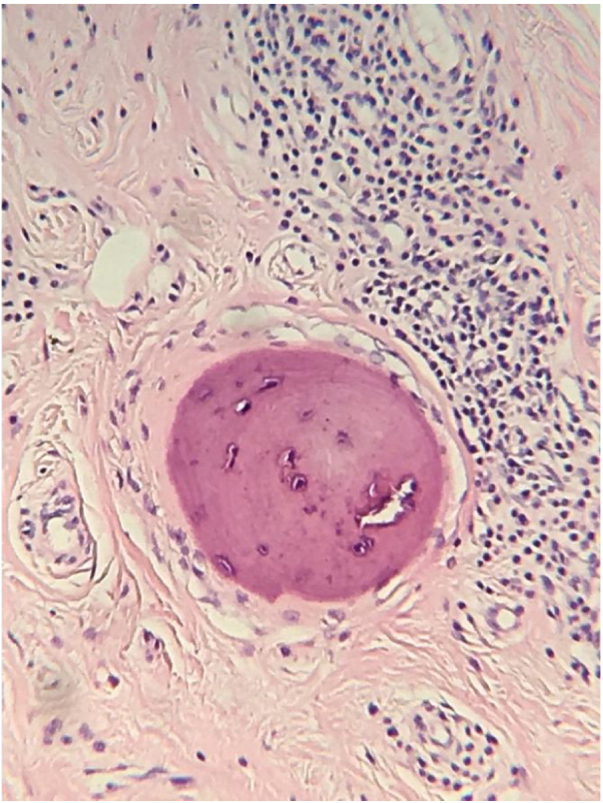
Fibroplasia, foreign body type granulomatous reaction around the free-haired shaft and bone formations compatible with cutaneous ossification Hematoxylin & eosin, x400).

**Figure 5:**
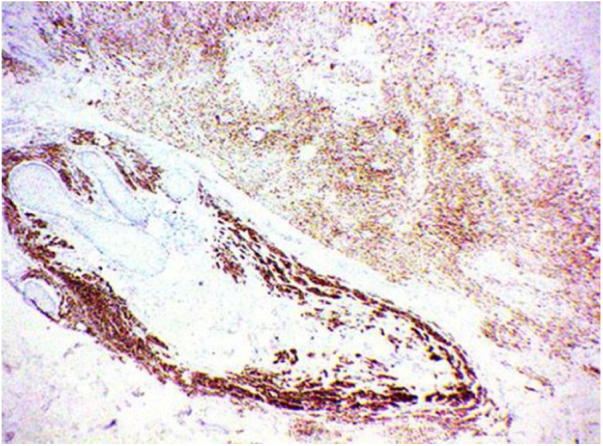
Immunohistochemical study demonstrating positivity for Melan A confirming the melanocytic nature of the lesion.

The histopathological analysis showed diffuse proliferation of melanocytes arranged in nests of regular size and shape in the papillary dermis and in strings in the reticular dermis surrounding adjacent structures with focal area of melanocytes of dendritic pattern with abundant melanic pigment in the cytoplasm. In addition to collagen fibroplasia, there was a foreign body type granulomatous reaction around free hair shaft and homogeneous bone formations compatible with cutaneous ossification. The histological diagnosis was osteonevus of Nanta in a combined nevus – intradermal and blue (Figure 3, Figure 4, Figure 5). The immunohistochemical study confirmed the melanocytic nature of the lesion, with positivity for the markers S-100, MART-1, gp100.

## Discussion

The first report of ossification in a melanocytic nevus was made in 1908 by Heidingsfeld, although the phenomenon was detailed published just in 1911 by Nanta, when it became known as osteonevus of Nanta.^2^

From its initial description, the phenomenon was reported in several melanocytic lesions such as blue nevus, Spitz's nevus,^5^ Becker's nevus,^6^ being the association with intradermal melanocytic nevus the most commom.^4^

According to the literature, the lesions are mostly located in the head and neck, especially on the face, being less than 5% in other locations, as forearms.^2^ It is more common in women,^2^, ^7^ and the age is variable, with a higher frequency in the elderly.^3^, ^4^ In the case reported, the location and sex of the patient are consistent with the literature data.

About its pathophysiology, disembrioplasia (hamartomatous lesion) and metaplasia are the main mechanisms postulated so far to explain the occurrence of the ossification of these lesions. According to the disembrioplasia, there would be the presence of primitive mesenchymal cells of osteocytes at erroneous sites with simultaneous expression of structures of ectodermal and mesodermal origin.^8^ In the metaplasia mechanism there would be chronic inflammation, trauma or neoplastic proliferation inducing differentiation of dermal fibroblasts into osteoblasts.^8^ In chronic inflammation/trauma a follicular distortion or obstruction caused by melanocytes or repetitive trauma of the follicle induced by the removal of hairs from the nevus would lead to follicular rupture, with consequent inflammation and metaplasia.^4^, ^8^ In the neoplastic processes desmoplasia would make the primitive dermal mesenchymal cells capable of producing bone.^9^ A third possibility would be that the simple interaction between nevus cells and mesenchymal tissue could result in cell metaplasia and consequent ossification.^3^, ^4^

Regarding the higher incidence of osteonevus in female reported in a large part of the studies, it is believed that estrogen has a potential role in bone formation.^4^ Since osteoblasts have receptors for estrogens, this interaction would result in their activation and blockade bone resorption by osteoclasts.^3^, ^10^ However, there is a lack of studies to prove the importance of this mechanism in the ossification process of melanocytic nevi.

In the histological analysis, ossification is usually observed as small islands of lamellar or amorphous compact bone at the base of the melanocytic lesion,^3^ in the reticular dermis near hair follicles,^4^, ^10^ as single or multiple focus.^3^ In some cases, bone trabecular lesions with bone marrow and adipocytes may be present.^3^ Mononuclear infiltrate, foreign body granuloma or mixed granulomas are frequent findings.^2^

In the case reported there was the characterization of combined nevus – intradermal melanocytic nevus and blue nevus – associated with the presence of homogeneous bone tissue in the reticular dermis near hair follicles and a foreign body type granulomatous reaction. No reports have been identified in the literature about combined nevus ossification, which is probably the first description of this association.

The findings of ossification near hair follicle and granulomatous reaction of the foreign body type, associated with the localization of the lesion in the scalp, site rich in hair follicles and target of repetitive trauma, guide the reasoning regarding the possibility of metaplasia resulting from a chronic follicular inflammatory process, leading to secondary ossification of the combined melanocytic nevus.

Culver and Burgdorf in 1993 reported the only case in the literature of malignant melanoma associated with osteonevus of Nanta. In the report, the female patient had extensive superficial melanoma and intradermal nevus with two ossification foci at its base.^9^ It is noted that there is a possibility of ossification in melanomas, but it occurs in the middle of tumor desmoplasia, different from the case reported by these authors that bone formation was at the basis of intradermal nevus.

Although the phenomenon does not appear to be related to a poor prognosis, the possibility of metaplasia as a pathophysiological mechanism and an association with melanoma^9^ become the osteonevo of Nanta a lesion with potential clinical importance. Therefore, the findings of ossification in excised melanocytic lesions should not be underestimated and not reported.

## Financial support

None declared.

## Author's contributions

Elaine Dias Melo: Conception and planning of the study; elaboration and writing of the manuscript; obtaining, analysis, and interpretation of the data; intellectual participation in the propaedeutic and/or therapeutic conduct of the studied cases; critical review of the literature.

Patrícia Amaral Couto: Elaboration and writing of the manuscript; obtaining, analysis, and interpretation of the data; critical review of the literature.

Antônio Pedro Mendes Schettini: Approval of the final version of the manuscript; elaboration and writing of the manuscript; effective participation in research orientation; critical review of the literature; critical review of the manuscript.

Carlos Alberto Chirano Rodrigues: Conception and planning of the study; effective participation in research orientation; intellectual participation in the propaedeutic and/or therapeutic conduct of the studied cases.

## Conflicts of interest

None declared.
